# Bilateral and Symmetrical Anteromedial Bowing of the Lower Limbs in a Patient with Neurofibromatosis Type-I

**DOI:** 10.1155/2015/425970

**Published:** 2015-02-28

**Authors:** Ali Al Kaissi, Klaus Klaushofer, Franz Grill, Rudolf Ganger

**Affiliations:** ^1^First Medical Department, Ludwig Boltzmann Institute of Osteology, Hanusch Hospital of WGKK and AUVA Trauma Centre Meidling, Hanusch Hospital, Vienna, Austria; ^2^Paediatric Department, Orthopaedic Hospital of Speising, Vienna, Austria

## Abstract

An 8-year-old girl was referred to our department because of generalized bowing of long bones (radii, ulnae, and femora) and significant bilateral and symmetrical posteromedial bowing of the tibiae and fibulae. The femora were laterally bowed whereas the tibiae and fibulae showed posteromedial bowing between the middle and distal thirds of the tibia with posterior cortical thickening effectively causing the development of bilateral congenital anterolateral bowing of the tibiae and fibulae. We referred to closing-wedge osteotomy of the left tibia along with fibular osteotomy in order to realign the deformity. Due to the delayed appearance of skin stigmata in her early life, the diagnosis of neurofibromatosis was ruled out. At the age of 9 years, café-au-lait spots and axillary freckling were apparent. Genetic tests confirmed von Recklinghausen disease (neurofibromatosis type-I (NF1)) (gene has been localised to 17q22). Interestingly, bilateral and symmetrical anteromedial bowing of the tibiae and fibulae has not been described in patients with NF-I.

## 1. Introduction

Bowing of the lower limbs is not an uncommon deformity in orthopaedic practice. Its occurrence might accompany a long list of intrinsic bone disorders and myogenic, neurogenic, and endocrinologic disorders. The differentiation of these conditions is the baseline in the management. Developmental bowing manifests varus angulation centred at the knee (metaphyseal beaking), thickening of the medial tibial cortices, and tilted ankle joints. Congenital bowing might manifest as posteromedial bowing with cortical thickening along the cavity of the curvature and, in some cases, diaphyseal broadening [1].

Neurofibromatosis type-I (NF-I) (von Recklinghausen) is an autosomal dominant disorder with main features of cutaneous pigmented lesions and multiple tumours arising from elements of the peripheral and central nervous system due to dysgenesis of the primitive ectoderm. The gene is located in chromosome 17 and its incidence is about 1 in 3000, though difficult to determine accurately since estimates tend to be based on series biased in favour of severe cases with gross cutaneous, neurological, or orthopaedic complications [2].

The many cases in which skin stigmata are the sole manifestations are largely overlooked except when identified during examination of relatives of an index case presenting with more serious problems [3]. The finding of café-au-lait patches on the skin of a child with failing vision or unilateral nystagmus makes the diagnosis of an optic nerve or chiasma glioma very likely. Optic pathway glioma is one of the most important associations in children with NF-I [4, 5].

There have been several studies on high tibial closing-wedge osteotomy but nevertheless there is great variability in the results, stemmed from the varied etiological understanding. Differences in patient outcome may be caused by wide heterogeneity among studies (e.g., different techniques and evaluation systems, varying degrees of deformity, and, most importantly, the underlying pathology of the deformity as in skeletal dysplasia) [4–6].

## 2. Case Report

An 8-year-old girl was referred to our department because of short stature and progressive bilateral and symmetrical angular deformity of the lower limbs. She was a product of uneventful gestation; at birth her length, weight, and OFC were around the 10 percentile. The mother was a 28 years old (gravida 3, abortus 0) married to a first related cousin. Her subsequent course of development has been of moderate retardation in acquiring the motor skills. The retarded motor development was correlated with obvious ligamentous hyperlaxity, and neuromuscular investigations have been carried out. Muscle biopsy and enzymatic investigation were normal. In addition, hearing, vision, and neurological tests were normal. Clinical examination at the age of 9 years showed recently developed axillary freckling and café-au-lait spots associated with tiny multiple subcutaneous neurofibromas compatible with NF-I.

The patient underwent a series of investigations, including complete blood cell counts, urine biochemistry, alkaline phosphatase, and renal function parameters and tests, aimed at investigating calcium, phosphorus, and vitamin D metabolism. However, all aforementioned tests were finally normal. Hormonal investigations included thyroid hormones, adrenocorticotropic hormone, and growth hormone which all were negative as well. Renal and pelvic ultrasounds were normal. Mapping of the NF-I gene was detected on chromosome 17 (the gene has been localised to 17q22).

**Figure 1:**
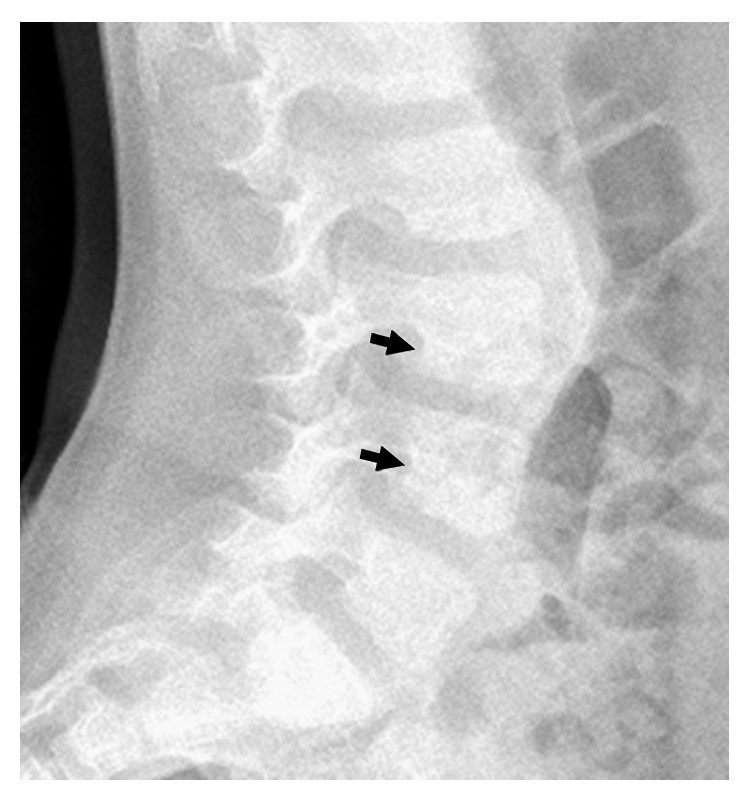
Lateral spine radiograph showed exaggerated lumbar lordosis associated with scalloping of the posterior end-plates (arrows).

**Figure 2:**
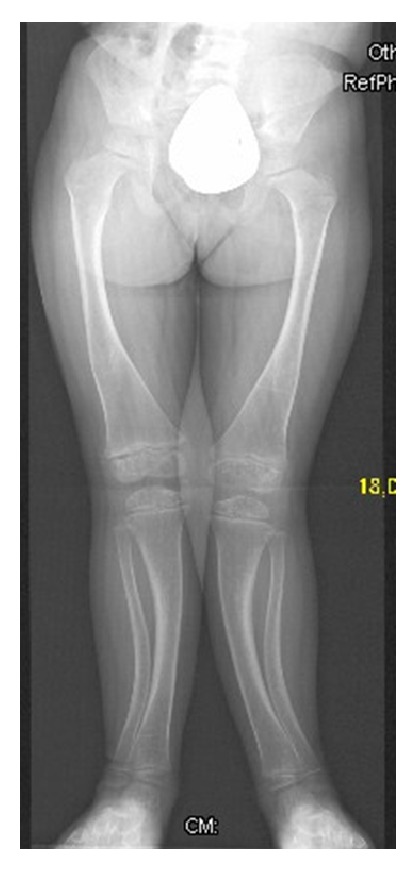
AP standing radiograph at the age of 7 years showed squaring of the iliac wings, coxa vara, and bilateral and symmetrical lateral bowing of the femora associated with thickening of the cortices. The tibiae and fibulae showed bilateral sabre deformity with posteromedial bowing between the middle and distal thirds of the tibia. Thickening of the tibial cortices was evident. The fibulae showed broadening/“tibialization” and posterior cortical thickening of the two bones.

**Figure 3:**
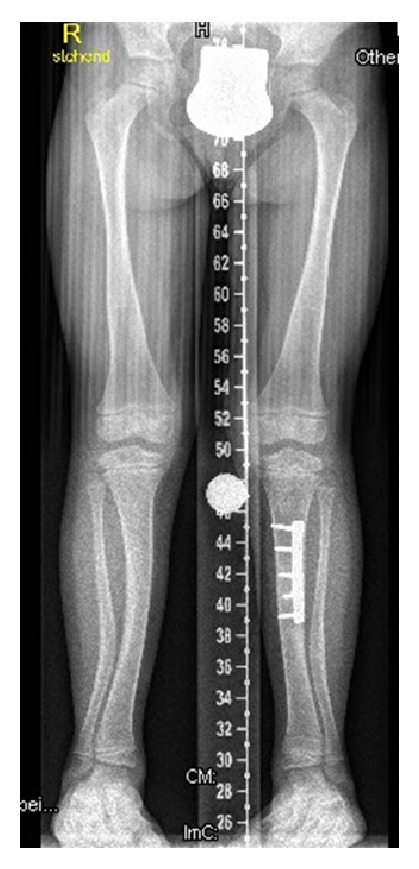
AP standing lower limb radiograph at the age of 11 years (after operation) showed lessening and improvement of the lower limbs deformity following the closing-wedge osteotomy of the left tibia and fibular-osteotomy.

Skeletal survey and lateral spine radiograph showed exaggerated lumbar lordosis associated with scalloping of the posterior end-plates (Figure 1). AP standing radiograph showed squaring of the iliac wings, coxa vara, and bilateral and symmetrical lateral bowing of the femora associated with thickening of the cortices. The tibiae and fibulae showed bilateral sabre deformity with posteromedial bowing between the middle and distal parts of the tibia. Thickening of the tibial cortices was evident. The fibulae showed broadening/“tibialization” and posterior cortical thickening (Figure 2). Treatment of the valgus deformity was performed with bilateral closed-wedge osteotomies using plate fixation. Because of the apex of the deformity at the diaphyseal area, we decided to perform the osteotomy and acute correction combined with internal fixation. In case of epi- or metaphyseal deformities usually we prefer temporary hemiepiphysiodesis using 8-plates. Postoperative standing lower limb radiograph showed lessening and improvement of the lower limbs deformity following the closing-wedge osteotomy of the left tibia and fibular-osteotomy (Figure 3).

## 3. Discussion

Neurofibromatosis type-I is a common autosomal dominant condition occurs in about 1 in 3000 individuals. The manifestations are protean and well known. The most common presenting features are cafe au lait patches (more than six greater than 1.5 cms in diameter) and peripheral neurofibroma [1]. Another factor that complicates diagnosis is that one half of cases represent new mutations. These sporadic cases of NF-I do not have affected first-degree relatives and are, therefore, more difficult to diagnose during early childhood before other features of the disease are apparent. Osseous lesions, such as sphenoid dysplasia, or thinning of the long bone cortex, usually present within the first year of life and occurs in around 14% of NF-I patients. Another factor that complicates diagnosis is that one half of cases represent new mutations. These sporadic cases of NF-I do not have affected first-degree relatives and are, therefore, more difficult to diagnose during early childhood before other features of the disease are apparent [1, 2, 4, 5].

There are three clinical forms of neurofibromatosis; first, is the peripheral form with café-au-lait spots and neurofibromas, second, the central from with multiple neoplasms in the central nervous system, and the third is the mixed form with both peripheral lesions and neurofibromas and tumours of the central nervous system [3]. There is a high incidence of skeletal involvement, the bone deformities resulting either directly from the destructive neurofibromatous tissue or from localized or systemic aberrations of skeletal growth and development. The most unusual and striking of these irregularities is focal gigantism, the result of hypertrophy of a single bone, digit, or entire limb. Overgrowth of an extremity is uncommon and usually unilateral. The hypertrophy may result from neurosegmental overgrowth, which implies that a nerve dysplasia rather than a primary bone dysplasia is responsible [7]. Crawford and Schorry, estimated that 5.7% of patients with NF-I develop anterolateral but not anteromedial bowing and they described the soft tissue changes in children with neurofibromatosis which include haemangiomatosis, lymphangiomatosis, elephantiasis, and numerous beaded plexiform neurofibromas [8]. Anterolateral tibial bowing is a morbid skeletal manifestation observed in 5% of children with neurofibromatosis type-I (NF-I), typically identified in infancy [9]. Stevenson et al. studied 23 cases of anterolateral bowings in NF-I in which radiographs were retrospectively reviewed. They encountered an excess of males (80%) with tibial bowing. The increase in male gender was primarily due to the group of individuals with complications of fracture, pseudarthrosis, surgery, and/or amputation, and co-workers have postulated that the excess number of males was due to increased activity leading to the subsequent complications. However, in this study, the retrospective review of radiographs excluded those individuals with tibial fracture, pseudarthrosis, or surgical manipulation. Therefore, it appears that male gender may be a risk factor for anterolateral bowing of the tibia. They considered hormonal influences could contribute to the development of tibial bowing [10–12].

Ferner et al., and Stevenson et al. reported 2–5% of patients with NF-I, showed long bone dysplasia, typically involved Long bone dysplasia in patients with NF-I, typically involves the tibia and frequently presents with anterolateral bowing that may progress to fracture and nonunion. Tibial dysplasia is most often unilateral, evident in the first year of life, and usually not associated with a neurofibroma at the site, suggesting a random molecular event. Because most pseudarthrosis of the tibia are not present at birth, the term congenital pseudarthrosis of the tibia is somewhat inaccurate, and dysplasia is the preferred term, but there is no question that the underlying disease process and deformation of the tibia are usually present at birth, and it is often merely a matter of time before first closure occurs [5]. Anterolateral bowing is not uncommon deformity in patients with neurofibromatosis type-I (NF-I). Up to 55% of cases of anterolateral bowing and pseudarthrosis are associated with neurofibromatosis. Other bone lesions may include defects of the cortices of bones (caused by irritation of the periosteum by neurofibromatous tissues); cyst-like rarefaction due to growth of the proliferating tissue within the medullary cavity leads to bowing, unusual shortening, and a change in the internal structure [4, 5, 10–13].

Osteotomies of the lower extremity carry a variable risk for delayed union, nonunion, infection, inadequate correction or overcorrection, compartment syndrome, and peripheral nerve injury, depending on the nature of osteotomy, the degree of deformity and correction, and other factors. These osteotomies may be opening wedge, closing wedge, or a combination of opening and closing. However, the risk of compartment syndrome after osteotomy of the tibia is higher with acute correction, and there is always some uncertainty when attempting to determine the exact amount of correction. Osteotomies are often performed for Blount disease, achondroplasia, and hypophosphatemic rickets and in patients with osteogenesis imperfect [14, 15]. The constellation of bilateral and symmetrical anteromedial bowing and thickening/broadening of the short and the long bones in our current patient have not been reported in patients with NF-I. The tibiae and fibulae showed medial convexity, and the apex of the curve was at the junction of middle and lower third. The trabecular pattern of the midshafts was somehow distorted and associated with posterior cortical thickening/hyperostosis. We preferred closed-wedge osteotomy because of the mid-diaphyseal apex of the deformity. In case of epi- and metaphyseal deformities usually we use guided growth with 8-plates. Finally, we wish to stress that the tibial abnormalities in our patient were evident very early in her life, whereas other findings that confirm the diagnosis of NF-I such as café-au-lait spots, axillary freckling, and cutaneous neurofibromas were not yet present but appeared later on.
